# Assigning biological function using hidden signatures in cystine-stabilized peptide sequences

**DOI:** 10.1038/s41598-018-27177-8

**Published:** 2018-06-13

**Authors:** S. M. Ashiqul Islam, Christopher Michel Kearney, Erich J. Baker

**Affiliations:** 1 Institute of Biomedical Studies, Baylor University, Waco, 76798 USA; 2 Department of Biology, Baylor University, Waco, 76798 USA; 3 Department of Computer Science, Baylor University, Waco, 76798 USA

## Abstract

Cystine-stabilized peptides have great utility as they naturally block ion channels, inhibit acetylcholine receptors, or inactivate microbes. However, only a tiny fraction of these peptides has been characterized. Exploration for novel peptides most efficiently starts with the identification of candidates from genome sequence data. Unfortunately, though cystine-stabilized peptides have shared structures, they have low DNA sequence similarity, restricting the utility of BLAST and even more powerful sequence alignment-based annotation algorithms, such as PSI-BLAST and HMMER. In contrast, a supervised machine learning approach may improve discovery and function assignment of these peptides. To this end, we employed our previously described m-NGSG algorithm, which utilizes hidden signatures embedded in peptide primary sequences that define and categorize structural or functional classes of peptides. From the generalized m-NGSG framework, we derived five specific models that categorize cystine-stabilized peptide sequences into specific functional classes. When compared with PSI-BLAST, HMMER and existing function-specific models, our novel approach (named CSPred) consistently demonstrates superior performance in discovery and function-assignment. We also report an interactive version of CSPred, available through download (https://bitbucket.org/sm_islam/cystine-stabilized-proteins/src) or web interface (**watson**.**ecs**.**baylor**.**edu/cspred)**, for the discovery of cystine-stabilized peptides of specific function from genomic datasets and for genome annotation. We fully describe, in the Availability section following the Discussion, the quick and simple usage of the CsPred website to automatically deliver function assignments for batch submissions of peptide sequences.

## Introduction

Cystine-stabilized peptides are impressively abundant and widespread across the taxa. They form the neurotoxic venom fraction of spiders^1^, snakes^2^, scorpions^3^, sea anemones^4^, jellyfish, corals and conch^5^ and may be specific for insects, mammals, or reptiles. Other cystine-stabilized peptides serve as antimicrobials^6^ and defensins in humans, insects, fungi, plants and most other taxa. Functionally, the venom peptides include sodium^7^, calcium^8^ and potassium^9^ ion channel blockers, acetylcholine receptor inhibitors^10^, or protease inhibitors^11^. Antimicrobial peptides generally act as membrane disrupters specifically against bacterial or fungal cells, but, due to their ability to penetrate cell membranes, they can also enter eukaryotic cells to act on host DNA directly and to modulate immune responses^6^. The stability of these peptides and their specific and powerful functions make them strong candidates for a variety of medical and agricultural applications, including pain relief, disruption of cancer development, and environmentally friendly insecticides, fungicides and bactericides, delivered either directly or via transgenes.

Cystine-stabilized peptides are also achieving commercial success. Clinically, alpha-bungarotoxin has a long history of use in isolating and identifying specific acetylchloline receptors and in the diagnosis of myasthenia gravis^10^. Aprotinin has been shown clinically effective against flu infection by inhibiting protease cleavage of HA0 to HA1 and HA2^12^, and Linaclotide is licensed for clinical use orally against irritable bowel syndrome^13^. The calcium channel blocker from conch, ziconotide (Prialt), is used clinically as a pain reliever^8^, and the chloride channel blocker from scorpion, chlorotoxin, reached Phase III trials as a treatment for glioblastoma cancer^14^. However, only a tiny fraction of cystine-stabilized peptides has been characterized experimentally^15–17^. To sort through the huge number of remaining cystine-stabilized peptides present in such a wide range of genomes for the purpose of classifying each of these peptides into one of the disparate functional groups, an efficient automated approach is warranted.

Sequence identity of the cystine-stabilized peptides varies broadly and can be distributed into different structural/motif and family-based (the native source of a peptide) classes^18^. The scorpion toxin-like superfamily^17,19,20^, agatoxins^21^, and conotoxins^22^ are examples of family-based classes, while STPs^23^, NTPs^23^, cyclotides^24^ and knottins^25^ are examples of structure or motif-based classes. Because of the high degree of heterogeneity in their primary sequences, several sequence alignment independent models have been reported to classify the structure of the cystine-stabilized /disulfide-rich family. For instance, Cypred^26^ predicts cyclic peptides including cyclotides; Knotter 1D predicts peptides with ICK motifs^27^; iCTX-Type structures predict types of Conotoxins targeting Ion Channels^28^; PredCSF predicts conotoxin superfamily from the primary protein sequences^29^; and PredSTP predicts sequential tri-disulfide motifs in cysteine rich peptide^23^. In addition, a specific functional group of cystine-stabilized peptides often come from different family or structural classes. Thus, family or structure/motif-based classification will may reveal the functional characteristic of a peptide. Under this context, it is necessary to develop a sequence alignment independent model to discover the functional characteristics in a family of origin or structure agnostic fashion.

Machine learning-based supervised models are widely used to predict the functional and structural class of proteins which are difficult to predict using sequence alignment-based algorithms. However, it is imperative to extract the relevant feature vectors (descriptors) and to implement an optimized classification algorithm to get expected performance from a model. Several classification algorithms have already been exploited to predict protein characteristics from the primary sequences^30–32^, but, extracting proper descriptors from protein sequences remains a challenging task. A number of descriptors, such as amino acid composition^33^, autocorrelation^34^, CTD (composition, transition, and distribution)^35^, conjoint triads^36^ and pseudo amino acid compositions^37^ are routinely used to build machine learning-based models. Recently, we demonstrated a complete pipeline of a classifier constructor where the feature generation model is integrated with a logistic regression algorithm^38^. This training set pipeline is denoted as m-NGSG (*modified n-gram* and *skip-gram*) where a modified *n-grams*^39^and *skip-grams*-based^40^ framework is used to generate descriptors from the protein sequences and utilize the hidden signatures from the descriptors for the supervised classification^41^. The m-NGSG framework has proven highly accurate for constructing reliable supervised prediction models^38^.

In this study, we applied m-NGSG to build five individual models to predict ion channel blockers, antimicrobial peptides, acetylcholine receptor inhibitors, serine protease inhibitors, and hemolytic proteins from disulfide stabilized proteins. Identification of hemolytic characteristics will allow the researcher to eliminate from consideration proteins cytotoxic to humans. The results demonstrate superiority of m-NGSG-based models to PSI-BLAST^42^, HMMER^43^ and other available models. Finally, we propose the CSPred model which combines the results of the five different models and gives a probability score for the five important functional characteristics of cystine-stabilized proteins. We also present three classifiers that assign ion channel blockers into three subclasses, sodium, potassium and calcium channel blockers.

## Material and Methods

### Data acquisition and preparation

The positive and negative datasets for ion channel blockers (ICB), antimicrobial peptides (AMP), acetylcholine receptor inhibitors (ACRI), serine protease inhibitors (SPI), and hemolytic proteins (HLP) are generated by obtaining protein sequences from UniprotKB (knowledgebase)^44^ using the search keys mentioned in Supplement Table 1. All the protein sequences, including positive and negative classes, contain a minimum of one disulfide bond and a chain size of less than 150 amino acid residues. Thereafter, the protein sequences are curated manually based on the functional attribute for each entry. A portion of the HLP positive dataset is collected from the HemoPI server^45^. Here, only the sequences containing a minimum of one pair of cysteines are selected from the dataset. The CD-HIT software^46^ is used to organize sequences based on identity thresh-holds to generate final datasets for each functional group of the cysteine stabilized proteins (See Supplement Table 1). From the positive and negative datasets of each selected functional group, 90% of the chains are retained for training sets, while 10% of the chains are reserved for out-of-sample test sets using a random shuffle-split process. The numbers of chains in each training and test sets are mentioned in Supplement Table 1. Further, to construct a separate compound model to classify the ICB into three different subclasses, we made three separate models using six different training sets (Supplement Table 2). The ICB classifier was constructed to classify the ICBs into sodium, potassium and calcium channel blockers. In order of make the process reproducible, the sequences of the training and test sets are provided in a separate supplementary folder (Supplementary Folder 1).

### Model construction using m-NGSG

**Figure 1:**
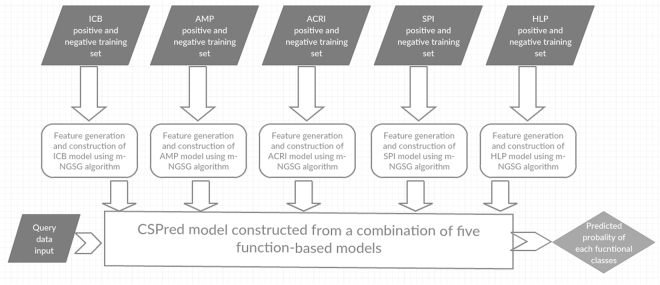
Work flow of CSPred construction and Implementation.

Five different binary classifiers are constructed to predict each of the five selected functional classes using the m-NGSG algorithm^38^. The m-NGSG algorithm (avialable at https://bitbucket.org/sm_islam/mngsg/src) offers an integrated and fully automated feature generation method followed by a logistic regression-based model construction, feature generation, and parameter optimization as described previously^38^. Parameter optimizations employed five-fold cross-validation using appropriate training sets. Supplement Table 3 illustrates the parameters selected by the m-NGSG optimizer for each functional group specific model. A combined model CSPred is further derived from the result aggregation of the five-individual function-based models. A diagram of the CSPred model construction is illustrated in Fig. 1.

### Model evaluation

The performances of all five models were evaluated using a five-fold cross-validation. Precision (eq. 1), recall (eq. 2), F1-score (eq. 3), accuracy (eq. 4), and Mathews Correlation Coefficient (MCC) (eq. 5) values are calculated for each model as the evaluation matrices. For calculation of these evaluation matrices, the confusion matrices were constructed to calculate the True Positives (TP), False Positives (FP), True Negatives (TN) and False Negatives (FN). TP and TN are correctly predicted positive and negative data points, respectively. Similarly, FP and FN are incorrectly predicted positive and negative data points, respectively. From TP, TN, FP, and FN, the evaluation matrices were calculated using the following equations:

1 $${\Pr }ecision=\frac{TP}{TP+FP}$$

2 $$\mathrm{Recall}{=}\frac{{TP}}{{TP}+{FN}}$$

3 $$F1\,score=\frac{2TP}{2TP+FP+FN\,}$$

4 $$Accuracy=\frac{TP+TN}{TP+TN+FP+FN\,}$$

5 $$MCC=\frac{(TP\times TN)-(FP\times FN)}{\sqrt{(TP+FP)(TP+FN)(TN+FP)(TN+FN)}}$$

### Comparison with PSI-BLAST and HMMER

The performance of each model except the subclasses of ICB is compared with PSI-BLAST^42^ and HMMER^43^. The ncbi-blast-2.5.0+ standalone software was downloaded to run PSI-BLAST locally. Similarly, HMMER 3.1b2 was installed in a Linux operating system, and the PHMMER function was used to run HMMER with the default parameters. Evaluation matrices were calculated for the identical training sets with PSI-BLAST using five-fold cross-validation. During cross-validation with PSI-BLAST and PHMMER, the training set was employed to populate the database, while the test set operated as the query. The class of each query sequence was predicted using the highest matching score with the sequences in the database. For PSI-BLAST, cross-validations were conducted using the threshold E-values of 0.01, 0.05, 0.1, 0.5, 1.0 and 5.0 with five iterations. All other parameters were kept as default. Afterwards, the sequence of the out-of-sample test sets from each functional group were predicted keeping the sequence of the corresponding training sets as databases.

### Comparison with other available models

Several other models exist to predict subsets of functional groups. iAMP-2L^47^ and CAMP_R_3^48^ are available to predict antimicrobial peptides, but are not scoped or optimized to predict cysteine stabilized peptides. We also compare the performance of our AMP model with iAMP-2L and CAMP_R_3. CAMP_R_3 offers four different classifiers to predict AMPs: Support Vector Machine (SVM), Random Forest (RF), Artificial Neural Network (ANN), and Discriminant Analysis (DA). We compared our AMP model with all classifiers offered by CAMP_R_3 using the out-of-sample test set and calculated precision, recall, F1-score, accuracy and MCC values. Similarly, the HemoPI^45^ model is dedicated to predicting hemolytic peptides, and was used to compare with our HLP model using the out-of-sample test set of HLP and calculated evaluation matrices.

## Results

### Evaluation of the m-NGSG-based models

**Figure 2:**
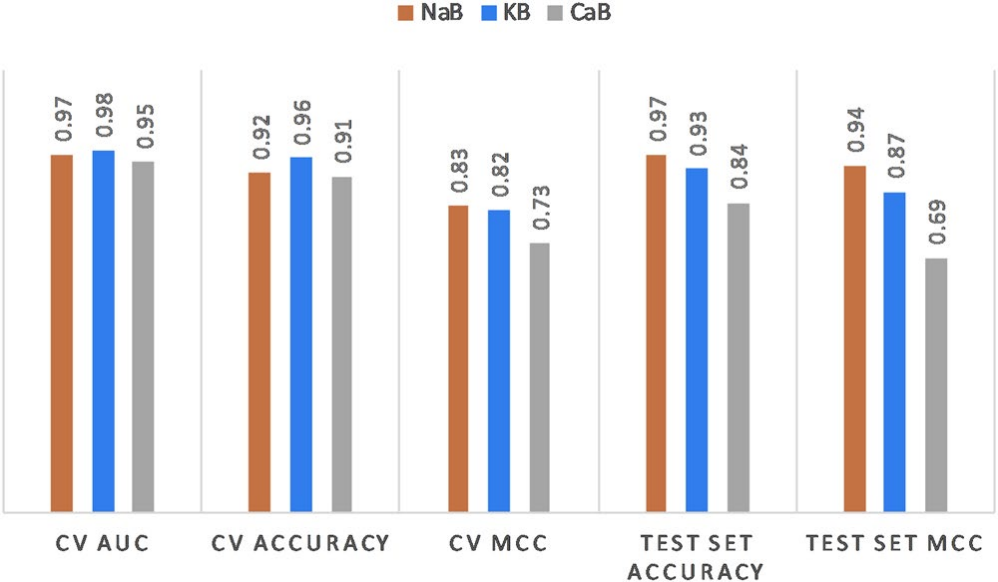
Performance of Ion Channel Blocker (ICB) sub-classifiers. NaB, KB and CaB represent the sodium, potassium and calcium channel blocker classifiers, respectively. CV AUC indicates the area under curve (AUC) using five-fold cross-validation; CV ACCURACY indicates the accuracy using five-fold cross-validation; CV MCC indicates the Mathews Correlation Coefficient (MCC) values using five-fold cross-validation; TEST SET ACCURACY indicates the accuracy using the out of sample test set; TEST SET MCC indicates the MCC values using the out of sample test set. These values indicate the robust performance of each classifier.

ICB, AMP, ACRI, SPI and HLP represent five different functional class-based models constructed using the m-NGSG algorithm^38^. Each model was evaluated using precision, recall, F1-score, accuracy and MCC scores based on a five-fold cross-validation against a training set. The evaluation matrices are reported in Table 1. The training set accuracies of the five models range from 86.33% to 95.23% where AMP and ACRI rendered the lowest and highest accuracy, respectively. The models also generated F1-Scores ranging from 0.81 to 0.89 and MCC scores ranging from 0.74 to 0.90. In addition, we observed consistent performances from all three ICB subclassifiers. The training set of the NaB, KB and CaB classifiers produced 0.83, 0.82 and 0.73 MCC scores, respectively, from five-fold cross-validations. These classifiers also generated MCC scores of 0.94, 0.87, 0.69, respectively which indicates trivial overfit/underfits of the models. Figure 2 illustrates the other evaluation matrices of the ICB subcalssification models.

**Table 1:** Comparison of evaluation matrices between the training and the out-of-sample test sets for each functional group-based model.

Models	Precision		Recall		F1-Score		Accuracy		MCC	
	Training set	Test set	Training set	Test set	Training set	Test set	Training set	Test set	Training set	Test set
ICB	91.25	95.58	83.80	92.85	0.87	0.94	89.67	95.32	0.78	0.90
AMP	86.56	85.96	77.08	81.66	0.81	0.84	86.33	87.74	0.71	0.74
ACRI	100.00	100.00	0.80	63.63	0.89	0.78	95.23	92.00	0.87	0.76
SPI	97.52	96.43	79.66	81.81	0.88	0.88	91.90	92.55	0.83	0.84
HLP	86.07	92.30	86.66	80.00	0.86	0.86	89.39	89.47	0.78	0.78

The precision, recall and accuracy values are shown in percentages.

Abbreviations: ICB = Ion channel blocker; AMP = Antimicrobial peptide; ACRI = Acetylcholine receptor inhibitor; SPI = Serine protease inhibitor; HLP = Hemolytic protein.

To judge the robustness of our approach, it is imperative to compare the performance of the model against established reliable available methods. PSI-BLAST and HMMER are used for generalized comparison, while other comparison groups are more specific. iAMP-2L and CAMP_R_3 are used to evaluate performance against AMPs, and HemoPI for HLP.

### Comparison of the evaluation matrices and area under curve (AUC) with PSI-BLAST and HMMER

**Figure 3:**
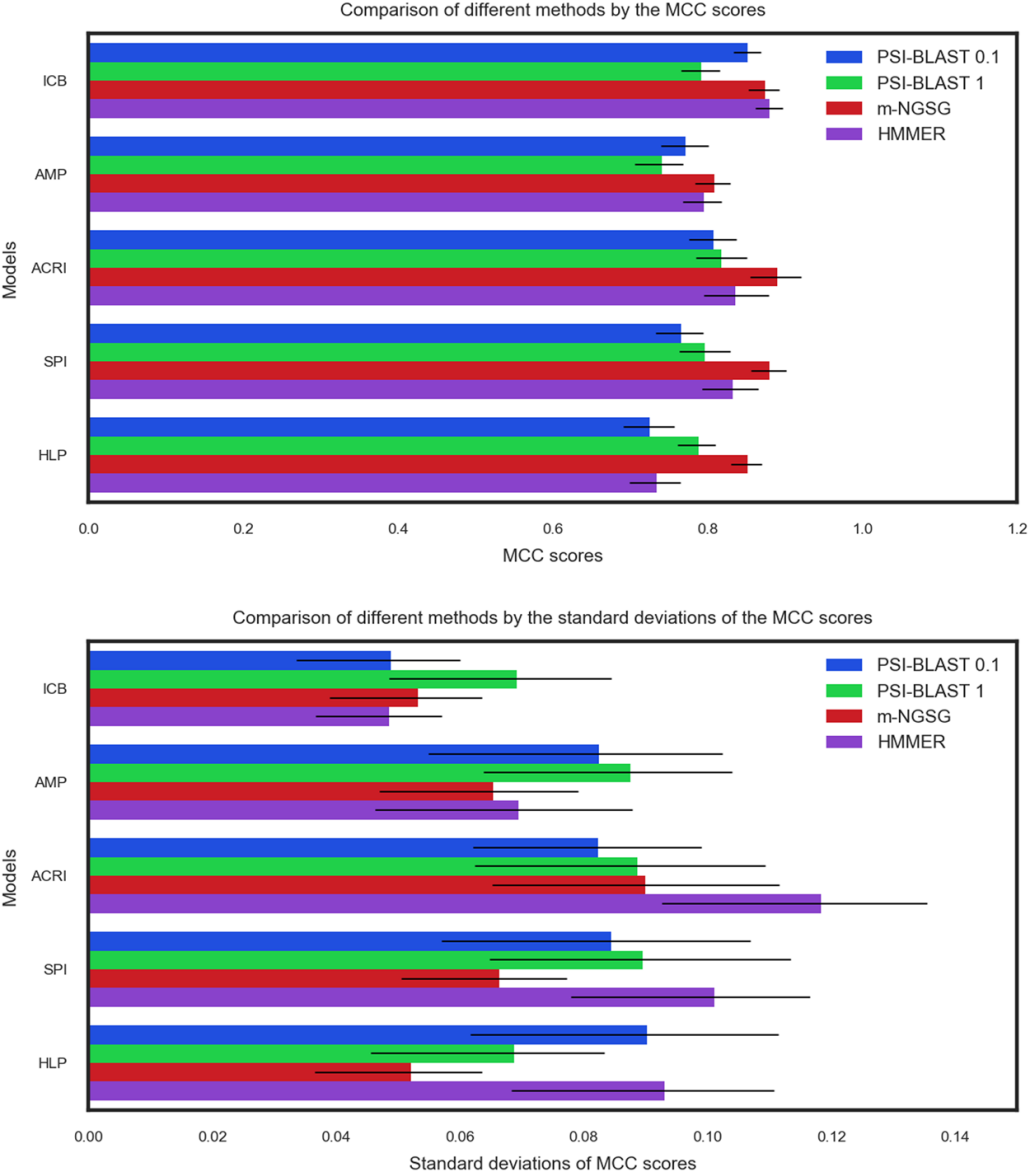
The depth of performance-consistency for each model. (**A**) (upper panel) illustrates the comparison of MCC (Mathews Correlation Coefficients) among PSI-BLAST (E-value 0.1 and 1), m-NGSG, and HMMER. The Y-axis indicates different function-based models; the X-axis indicates the MCC values with their standard errors. Each bar plot depicts the method used to build the models. (**B**) (lower panel) illustrates the comparison of standard deviations of MCC (Mathews Correlation Coefficients) scores among PSI-BLAST (E-value 0.1 and 1), m-NGSG, and HMMER. The Y-axis indicates different function-based models; the X-axis indicates the standard deviations of the MCC values with their standard errors. Each bar plot depicts the method used to build the models. Here, the higher the standard deviation, the lower the performance-consistency. The m-NGSG-based models show standard deviations of MCC values lower than 0.05 for each model while HMMER and PSI-BLAST return high standard deviations on some models. Please see Supplement Figs S2 and S3 for more details.

**Figure 4:**
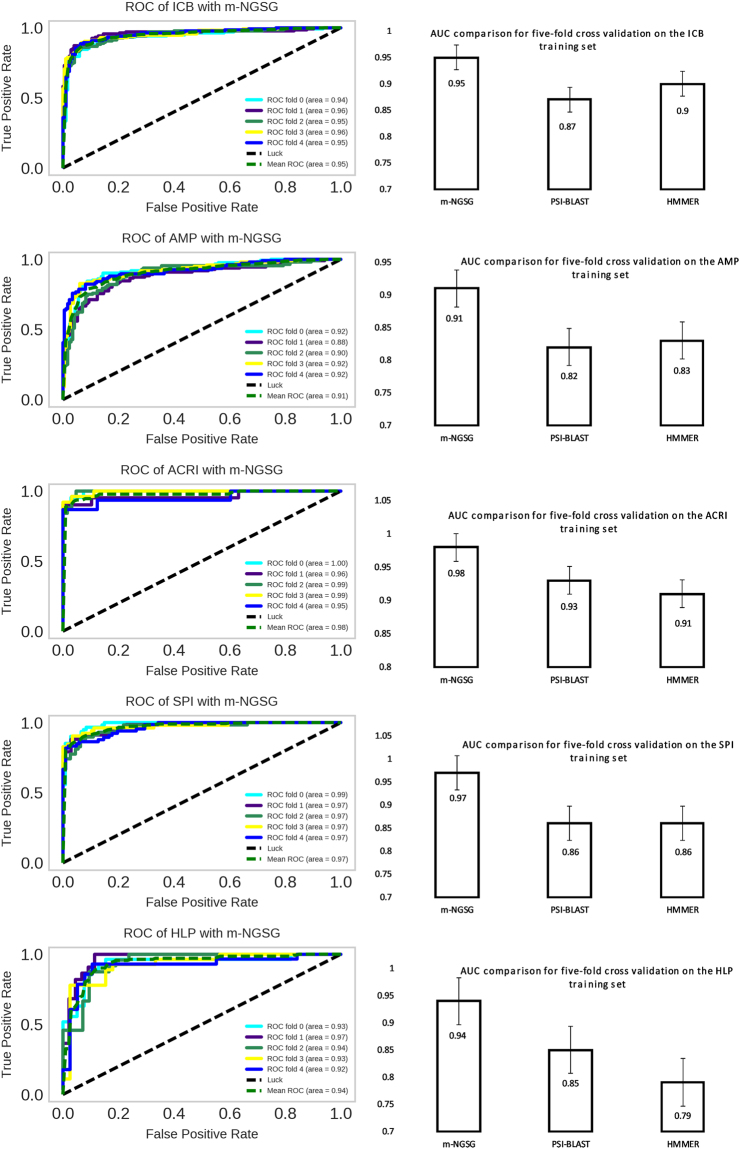
AUC among m-NGSG, HMMER and PSI-BLAST with best scoring MCC value. The left panel indicates the receiver operating characteristics of m-NGSG-based models. The right panel indicates the comparison of AUC among m-NGSG, PSI-BLAST, and HMMER for the corresponding function-based model. The height of each bar represents the AUC for each method. m-NGSG-based models demonstrates better AUC than PSI-BLAST and HMMER.

PSI-BLAST is a dependable and widely used algorithm to discover distantly related protein sequence using PSSM matrices^42^. HMMER is a Hidden Markov Model-based algorithm designed to detect remote homologs with a high sensitivity^43^. We compared the performance of each constructed model with PSI-BLAST and HMMER for the corresponding training sets using a five-fold cross-validation. Supplement Fig. 1 illustrates an extensive comparison among the m-NGSG based models, HMMER, and PSI-BLAST models made with different E-values. Precision, recall, F1-score, accuracy and MCC values are used to evaluate the models against PSI-BLAST. Figure 3A and Supplement Fig. 3 specifically show the comparison of the MCC values of each training set with PSI-BLAST and HMMER. Figure 3B and Supplement Fig. 3 illustrates the standard deviation of the MCC values generated from different folds using different models. The area under curve (AUC) for the five-different m-NGSG-based models were also compared with PSI-BLAST and HMMER using the corresponding training sets. The E-values yielding the best MCC values for each function-based training sets were used to run a PSI-BLAST for the comparison. Figure 4 shows the receiver operating characteristic (ROC) curves for m-NGSG-based models with a side by side area under curve (AUC) comparison among each m-NGSG-based model and the corresponding PSI-BLAST and HMMER-based models. For the five training sets, m-NGSG based models generated better AUCs compared to the corresponding PSI BLAST and HMMER based models.

### Comparison of the evaluation matrices with PSI-BLAST and HMMER on the out-of-sample test set

**Figure 5:**
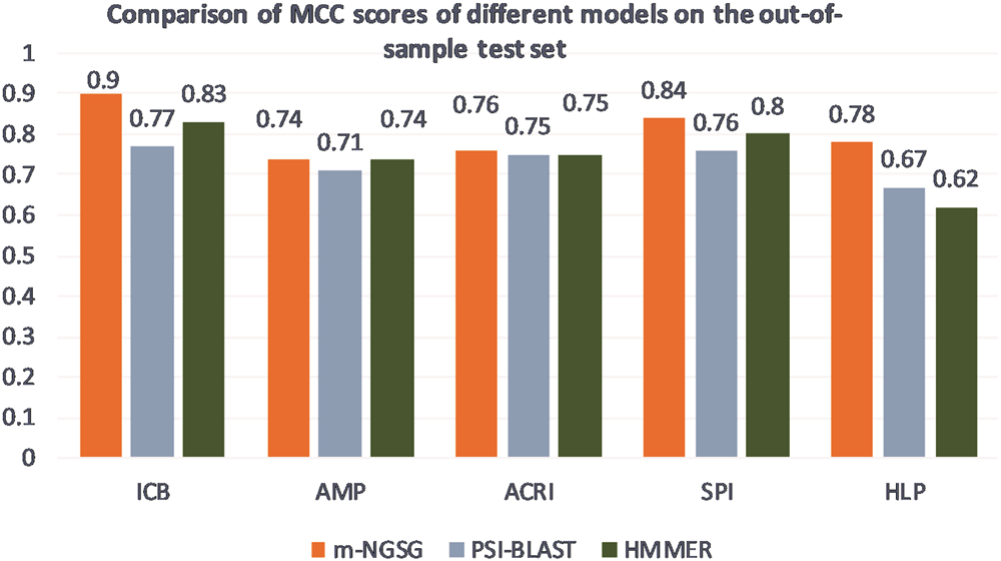
Comparison of MCC values on the out-of-sample test set with function-based model using m-NGSG, PSI-BLAST, or HMMER. While the MCC scores of HMMER are comparable for the AMP and ACRI test tests, the MCC score for ICB, SPI and HLP are noticeably lower compared to the m-NGSG-based models.

Versatility of the five m-NGSG based models were tested by comparing their performance with PSI-BLAST and HMMER on the corresponding out-of-sample test set. We imported the same E-values from the ROC curve comparison to run the PSI-BLAST on the test sets. The MCC values were measured for each model and the corresponding PSI-BLAST and HMMER to achieve an appropriate comparison. Figure 5 displays comparative bar plots which illustrate the MCC values on the out-of-sample test set produces five different models and PSI-BLAST. According to Fig. 5, each of the five models shows better MCC values compared to their equivalent PSI-BLAST results while four models show better MCC value than HMMER. In the case of AMP, both the m-NGSG-based model and HMMER returns the same MCC value (0.74).

### Comparison of AMP and Hemolytic peptide prediction models with other currently available models

**Figure 6:**
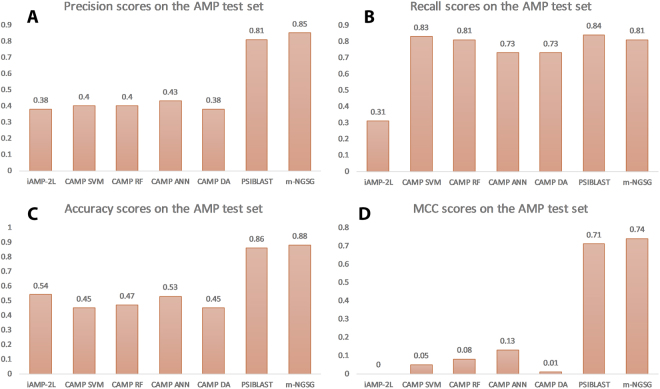
Precision, Recall, Accuracy and MCC. The precision, recall, accuracy and MCC values obtained applying iAMP-2L, CAMP SVM, CAMP RF, CAMP ANN, CAMP DA, PSI-BLAST E-value 0.1 and AMP (m-NGSG-based AMP model) on the out-of-sample AMP test set. (**A**) illustrates that precision values of iAMP-2L and CAMP models are considerably lower than the m-NGSG based model. (**B**) illustrates that the recall values of the CAMP models are comparable to the m-NGSG-based model while iAMP-2L demonstrates a noticeably lower recall value. (**C,D**) shows considerably low MCC and accuracy values displayed by iAMP-2L and CAMP models compared to PSI-BLAST and m-NGSG.

Along with PSI-BLAST and HMMER, we used the iAMP-2L^47^ and CAMP_R_3^48^ models to predict antimicrobial peptides (AMP), and the HemoPI^45^ algorithm to predict hemolytic peptides. While it is important to note that none of these models are dedicated to the identification of only cystine- stabilized peptides, their performance parameters should generalize to their prediction. We compared performances of iAMP-L2 and CAMP_R_3 with our m-NGSG-based AMP model and HemoPI with the m-NGSG-based HLP model using the corresponding out-of-sample test sets. Figure 6 shows the comparative precision, recall, accuracy and MCC values among different models. Among the other available models, CAMP-ANN showed the highest precision score 0.43 or 43% while the precision score produced by m-NGSG-based AMP model was 0.85. CAMP-SVM showed a slightly better recall score than m-NGSG, 0.83 and 0.81, respectively. Overall, the best accuracy score was generated by iAMP-2L (0.54), but it was far less than the accuracy score produced by m-NGSG, which was 0.75. Finally, the highest MCC score was generated by CAMP-ANN (0.13) which was also well below the MCC score of m-NGSG (0.74) (see Fig. 6). Similarly, Supplement Fig. 4 illustrates the comparison on the out-of-sample HLP test set among Hemo PI, PSI-BLAST and m-NGSG. Here, precision, recall, accuracy and MCC scores of HemoPI are 0.55 (55%), 0.67 (66%), 0.66 (66%) and 0.31, respectively. These are lower than corresponding scores of m-NGSG-based HLP.

## Discussion

In this study, we constructed five different functional classifiers of cystine -stabilized peptides and combined them to build the CSPred model which predicts the probability of the selected five functional characteristics of query peptide sequences. After building a model, the most important step is to assess its performance using a k-fold cross-validation and out-of-sample test sets. We have performed this step carefully using a five-fold cross-validation and an out-of-sample test set for each of the five models. Table 1 shows a comprehensive comparison among evaluation matrices for each model. The cross-validation accuracy ranged from ~86% to ~95% for the models. The accuracies on the out-of-sample test sets were concordant to the cross-validation accuracies. No big difference was detected between the accuracies for the models except the ICB model where the difference between the test and training set was ~5%. However, the increase of accuracy on the test set indicates versatility of the ICB model. The other evaluation matrices such as F1-score and MCC were also quite consistent between the training and test set (see Table 1). The comparative analysis between the evaluation matrices explains the adaptability of each different function-based model.

The protein sequences collected from the Uniprot Knowledgebase were further filtered using the CD-hit^46^ software to reduce the homoscedasticity of the model by reducing the redundancy of the similar sequences. Although this operation reduced the amount of available training set, particularly of the ICB subclasses, that helped models to be kept optimally unbiased. The fasta sequences of each protein of the training and test sets from Uniprot Knowledgebase may contain some other domains along with the functional domains. Despite the presence of these noises (the subsequences from other domains) in the datapoints (here the data points are the primary sequences of the proteins), CSPred showed a robust performance in the cross-validation and out-of-sample test-set classification. This outcome indicates the strength of m-NGSG algorithm to classify data points with noise. From a previous study, we demonstrated that m-NGSG is capable to differentiate noises in datasets during a classification task^38^.

The ultimate success and novelty of a machine learning-based model depends on its superiority over other concurrent algorithms. PSI-BLAST and HMMER are successful and widely accepted algorithms to discover distantly related protein chains. Therefore, we compared the performance of each five function-based model with PSI-BLAST and HMMER using the corresponding training and out-of-sample test sets. One complexity and disadvantage to working with PSI-BLAST is choosing the optimal E-value; it is challenging to select an E-value that will give the best results. Supplement Fig. 1 shows a clear superiority of m-NGSG-based methods over the equivalent PSI-BLAST with different E-values and HMMER except better recall values obtained by HMMER compared to m-NGSG based models. That explains only a better sensitivity of HMMER than m-NGSG models but not the overall performance. The MCC score is chosen over an accuracy score for further comparison because MCC is more robust and reflects the sensitivity, specificity, precision and false negative rate while accuracy only reflects the average of sensitivity and specificity of a model^49^. Similar to the training set, m-NGSG-based models showed better MCC values on the out-of-sample test sets compared to the corresponding HMMER and PSI-BLAST with the optimized E-values, Fig. 5. This result demonstrates consistently better performance over HMMER and PSI-BLAST and unbiased behavior of the m-NGSG-based models. In addition to PSI-BLAST and HMMER, we compared the m-NGSG-based model with other available function specific prediction models. There are two available models to predict antimicrobial peptides: iAMP-2Land CAMP_R_3. CAMP_R_3 also has four different classifiers to perform the antimicrobial peptide prediction which are SVM, RF, ANN, and DA. We evaluated all the models by computing the four evaluation matrices (described in Fig. 5) on the out-of-sample test sets. The performance of iAMP-2L and CAMP_R_3 were significantly low compared to PSI-BLAST and m-NGSG-based AMP model. The reason is possibly the training sets are not optimized to predict the cystine-stabilized AMPs. The similar results are found when we compared HemoPI (see Supplement Fig. 4) with the m-NGSG-based HLP model. These results indicated that the m-NGSG-based models are superior to any other concurrent algorithms to classify functions of cystine-stabilized peptides.

Several cystine-stabilized peptides have already been licensed for clinical or agricultural use. This small fraction demonstrates the potential for new applications hidden among the thousands of undiscovered cystine-stabilized peptide sequences in genomes across many taxa. A voltage-gated calcium channel blocker cystine-stabilized peptide (Hv1a) from spider venom^50^ is now the primary product of Vestaron, Inc., with commercial production in *E. coli* for broad-scale application on crops plants as an eco-friendly insecticide that degrades within two weeks after application. This same spider peptide has been fused to a targeting moiety by another group to specifically target aphids as a transgene in plants^51^. In our own lab (CMK), antimicrobial cystine-stabilized peptides have been targeted for specific toxicity towards individual pathogenic bacterial species, with nontarget toxicity greatly reduced (Islam *et al*., unpublished data). This has implications for antibiotic treatment without the disruption of the native microbiome. Thus, a diverse array of different cystine-stabilized peptides has realized commercial application.

The model approach outlined here has the potential to greatly impact the discovery of functionally active peptides. A typical pipeline might involve finding top candidate peptide sequences using genome databases and one or more of the prediction models, followed by peptide production of the top candidates in an appropriate heterologous expression system and wet lab evaluation of the peptides. Using an antimicrobial peptide screen as an example, our AMP algorithm would be used to screen a dataset of peptides (see the Availability section below). These candidate sequences would then be screened with the HLP (hemolytic protein) algorithm to eliminate peptides that might be toxic to human cells. From the pool of remaining candidates, synthesized DNA sequences would be cloned into an *E*. *coli* expression vector alongside a stabilizing fusion partner such as SUMO^52^ and a purification tag such as 6x His-Tag. The peptides would be expressed and purified, and then confirmed for stability, toxicity against the target microbe, and lack of toxicity against human cells. Top candidates would be available for pharmaceutical production systems or to be used as transgenes in the organism to be protected by the antimicrobial peptide^53^. An advantage with using peptides over typical small molecule drugs is the relative ease with which an appropriate modifying peptide can be found and genetically fused to the effector peptide sequence, for example an antimicrobial peptide targeted to a specific pathogenic bacterium with a targeting peptide^54^. It should be noted that the metadata associated with the peptide candidate sequence can also be used to help select peptides with the desired action. For example, ion channel blockers with oral toxicity might be more commonly found in plants and algae, where an ion channel blocker might serve as an oral insecticide, than in spider or snake venom, where the ion channel blocker would most likely have toxicity only by injection.

### Availability

CSPred is an open source collaborative initiative available in the bitbucket repository (https://bitbucket.org/sm_islam/cystine-stabilized-proteins/src). It is also publicly available as a free web application at watson.ecs.baylor.edu/cspred. The web server provides an accessibility to the CSPred, and a user does not need computational experience to use the model. Posting the web address (watson.ecs.baylor.edu/cspred) on a web browser will take the user to the CSPred webpage. There, the user needs to upload the fasta file of the unknown protein sequences and click the submit button. That action will trigger the prediction process. The result is divided into six columns. The first column is the protein ID labels of the fasta sequences. The second, third, fourth, fifth, and sixth columns display the probability values of being an ICB, AMP, ACRI, SRI, and HLP, respectively, for each sequence submitted. Thus, the web interface provides a simple avenue to categorize submitted protein sequences according to these five functional characteristics, and may use a high-throughput batch-style input. Supplementary Figs 5–7 demonstrate the pipeline to use the CSPred web application. The sub classifiers of ICB are not included in CSPred. However, all the training and test datasets are provided as a supplement enabling users to make their own models or reproduce the same models using the m-NGSG framework that is available at watson.ecs.baylor.edu/ngsg.

## Electronic supplementary material

Supplementary Info

Supplementary Dataset 1

Supplementary Dataset 2

Supplementary Dataset 3

Supplementary Dataset 4

Supplementary Dataset 5

Supplementary Dataset 6

Supplementary Dataset 7

Supplementary Dataset 8

Supplementary Dataset 9

Supplementary Dataset 10

Supplementary Dataset 11

Supplementary Dataset 12

Supplementary Dataset 13

Supplementary Dataset 14

Supplementary Dataset 15

Supplementary Dataset 16

Supplementary Dataset 17

Supplementary Dataset 18

Supplementary Dataset 19

Supplementary Dataset 20

Supplementary Dataset 21

Supplementary Dataset 22

Supplementary Dataset 23

Supplementary Dataset 24

Supplementary Dataset 25

Supplementary Dataset 26

Supplementary Dataset 27

Supplementary Dataset 28

Supplementary Dataset 29

Supplementary Dataset 30

Supplementary Dataset 31

Supplementary Dataset 32
