# Supplementary material for: Assigning biological function using hidden signatures in cystine-stabilized peptide sequences
Source: Sci Rep. 2018 Jun 13;8:9049. doi: 10.1038/s41598-018-27177-8 (PMC5998126; doi:10.1038/s41598-018-27177-8)
Supplement: Supplementary file 1 — Supplementary Info [file 41598_2018_27177_MOESM1_ESM.docx]

**Assigning biological function using hidden signatures in cystine -stabilized peptide sequences**

S M Ashiqul Islam^1^, Christopher Michel Kearney^1,2^ and Erich J Baker^1,3,*^

^1^ Institute of Biomedical Studies, Baylor University, Waco, 76798, USA

^2^ Department of Biology, Baylor University, Waco, 76798, USA

^3^ Department of Computer Science, Baylor University, Waco, 76798, USA

* To whom correspondence should be addressed. Tel: +1 01 254 710-3876; Fax: +1 01 254 710-3889; Email: [Erich_Baker@Baylor.edu](mailto:Erich_Baker@Baylor.edu)

Present Address: Erich Baker, Department of Computer Science, Baylor University, Waco, TX, 76798, USA

**Supplement Table 1**. Dataset description for each cystine stabilized functional group, their sequence similarity and number of chains used for training and test sets.

| Dataset | | UniProtKB (protein knowledgebase) search key | Sequence identity*<* | No. of chains | No. of  chains  in the  training set | No. of chains in the out of sample test  set |
| --- | --- | --- | --- | --- | --- | --- |
| ICB | Positive | channel toxin annotation:(type:disulfid) length:[1 TO 150] NOT annotation:(type:function acetylcholine) NOT hormon (sodium OR nav OR potassium OR kv OR calcium  OR cav OR ion) AND reviewed:yes | 70% | 697 | 627 | 70 |
|  | Negative | NOT nav NOT cav NOT kv NOT sodium NOT potassium NOT calcium NOT ion (defensin OR acetylecholine OR serine OR hormon OR enzyme OR antimicrobial OR bateria) length:[1 TO 150] annotation:(type:disulfid) AND reviewed:yes | 70% | 1004 | 903 | 101 |
| AMP | Positive* | (defensin OR antimicrobial OR antibacterial OR antifungal OR antiviral) NOT ”defensin-like” length:[1 TO 150] annotation:(type:disulfid) AND reviewed:yes | 70% | 611 | 551 | 60 |
|  | Negative | NOT defensin NOT antimicrobial NOT antibacterial NOT antifungal NOT antiviral NOT defense (channel OR ion) length:[1 TO 150] annotation:(type:disulfid) AND reviewed:yes | 70% | 945 | 850 | 95 |
| ACRI | Positive | (acetylcholine OR achr OR nachr) channel NOT nav NOT kv NOT cav NOT sodium NOT potassium NOT calcium  length:[1 TO 150] annotation:(type:disulfid) | 50% | 105 | 94 | 11 |
|  | Negative | channel toxin (defensin OR nav OR kv OR cav OR sodium OR potassium OR calcium OR agouti OR serine OR enzyme) NOT acetylcholine NOT achr NOT nachr NOT  snake length:[1 TO 150] annotation:(type:disulfid) | 90% | 381 | 342 | 39 |
| SPI | Positive | serine protease inhibitor annotation:(type:disulfid) length:[1 TO 150] NOT defensin NOT antimicrobial NOT antifungal NOT acetylcholine NOT nav NOT kv NOT cav NOT sodium NOT potassium NOT calcium NOT channel AND reviewed:yes | 90% | 328 | 295 | 33 |
|  | Negative | NOT serine NOT protease annotation:(type:disulfid) length:[1 TO 150] (defensin OR antimicrobial OR antifungal OR acetylcholine OR nav OR kv OR cav OR sodium OR potassium OR calcium OR channel) AND reviewed:yes | 40% | 604 | 543 | 61 |
| HLP | Positive* | (hemolytic OR cytolytic OR cytoxic) annotation:(type:disulfid) length:[1 TO 150] annotation:(type:function hemolytic) NOT annotation:(type:function weak) NOT ”no hemolytic” | 90% | 146 | 131 | 15 |
|  | Negative* | annotation:(type:disulfid) length:[1 TO 150] NOT annotation:(type:function hemolytic) NOT hemolytic annotation:(type:function toxin) NOT mammalia NOT mammalian NOT cytotoxic NOT cytolytic NOT snake NOT name:”alpha mammalia” NOT name:”beta mammalia” AND reviewed:yes + (”no hemolytic” OR ”non hemolytic” OR ”not have hemolytic” OR ”lack hemolytic”) annotation:(type:disulfid) length:[1 TO 150] AND reviewed:yes | 50% | 222 | 199 | 23 |

Abbreviations: ICB = Ion channel blocker; AMP = Antimicrobial peptide; ACRI = Acetylcholine receptor inhibitor; SPI= Serine protease inhibitor; HLP = Hemolytic protein

* The AMP positive training set was manually curated after performing the sequence identity-based clustering step. The positive and negative training set of HLP were collected using composite search followed by combining two of more datasets. Also, the positive training set of HLP was manually curated to filter noise.

**Supplement Table 2**. Dataset description for each subclass within ICB, their sequence similarity and number of chains used for training and test sets. NaB, KB and CaB represents the sodium, potassium and calcium channel blocker classifiers, respectively.

| Dataset | | Sources of Datasets | Sequence identity*<* | No. of chains | No. of  chains  in the  training set | No. of chains in the out of sample test  set |
| --- | --- | --- | --- | --- | --- | --- |
| NaB | Positive | Annotated as sodium channel blockers (Nab) but not potassium (Kb) or calcium (Cab) channel blockers in the Uniprot database | Nab 90% | 379 | 341 | 38 |
|  | Negative | Annotated potassium (Kb) or calcium channel blockers (Cab) but not sodium channel blockers (Nab) in the Uniprot database | Kb 65% and Cab 90% | 302 | 271 | 31 |
| KB | Positive | Annotated as potassium channel blockers (Kb) but not sodium (Nab) or calcium channel blockers (Cab) in the Uniprot database | Kab 90% | 286 | 257 | 29 |
|  | Negative | Annotated sodium (Nab) or calcium channel blockers (Cab) but not potassium channel blockers (Kb) in the Uniprot database | Nab 65% and Cab 90% | 322 | 289 | 33 |
| CaB | Positive | Annotated as calcium channel blockers (Cab) but not sodium (Nab) or potassium channel blockers (Kb) in the Uniprot database | Cab 95% | 147 | 132 | 15 |
|  | Negative | Annotated sodium (Nab) or potassium channel blockers (Kb) but not calcium channel blockers (Cab) in the Uniprot database | Nab 65% and Kab 65% | 167 | 150 | 17 |

**Supplement Table 3**. Selected m-NGSG parameters for each model. Description of each parameter is discussed in detailed in *Islam et al*, 2017^1^.

| Training set |  | n | k | np | kp | y | c |
| --- | --- | --- | --- | --- | --- | --- | --- |
| ICB |  | 7 | 13 | 1 | 7 | 10 | 4 |
| AMP |  | 4 | 19 | 1 | 1 | 3 | 1 |
| ACRI |  | 3 | 1 | 1 | 1 | 2 | 1 |
| SPI |  | 3 | 10 | 1 | 7 | 5 | 1 |
| HLP |  | 1 | 1 | 1 | 1 | 3 | 1 |
| NaB |  | 1 | 6 | 1 | 1 | 5 | 1 |
| KB |  | 4 | 16 | 1 | 1 | 5 | 1 |
| CaB |  | 1 | 3 | 1 | 1 | 5 | 1 |


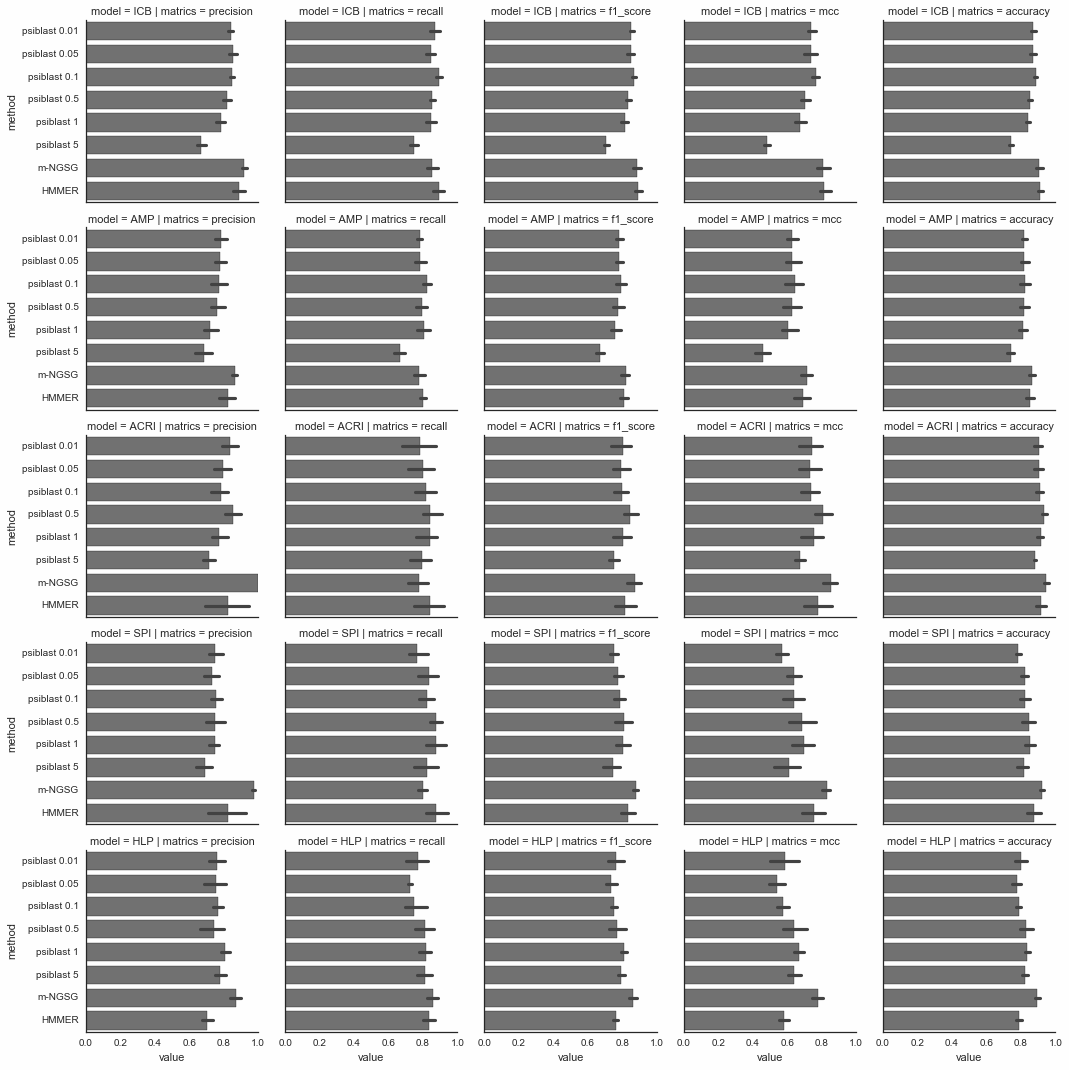


**Supplement Figure 1: Performance comparison of each classification method for different functional-based models using five-fold cross-validation.** Each row in the facet grid plot represents the function-based models while each column represents an evaluation matric. The Y-axis of each bar plot show the different methods used to build a model and the X-axis shows the values of the evaluation matrices with error bars. Except the ICB dataset, m-NGSG-based models return better F1-scores, MCC and accuracy values for all other four datasets, while PSI-BLAST returns lower values for all the data sets.


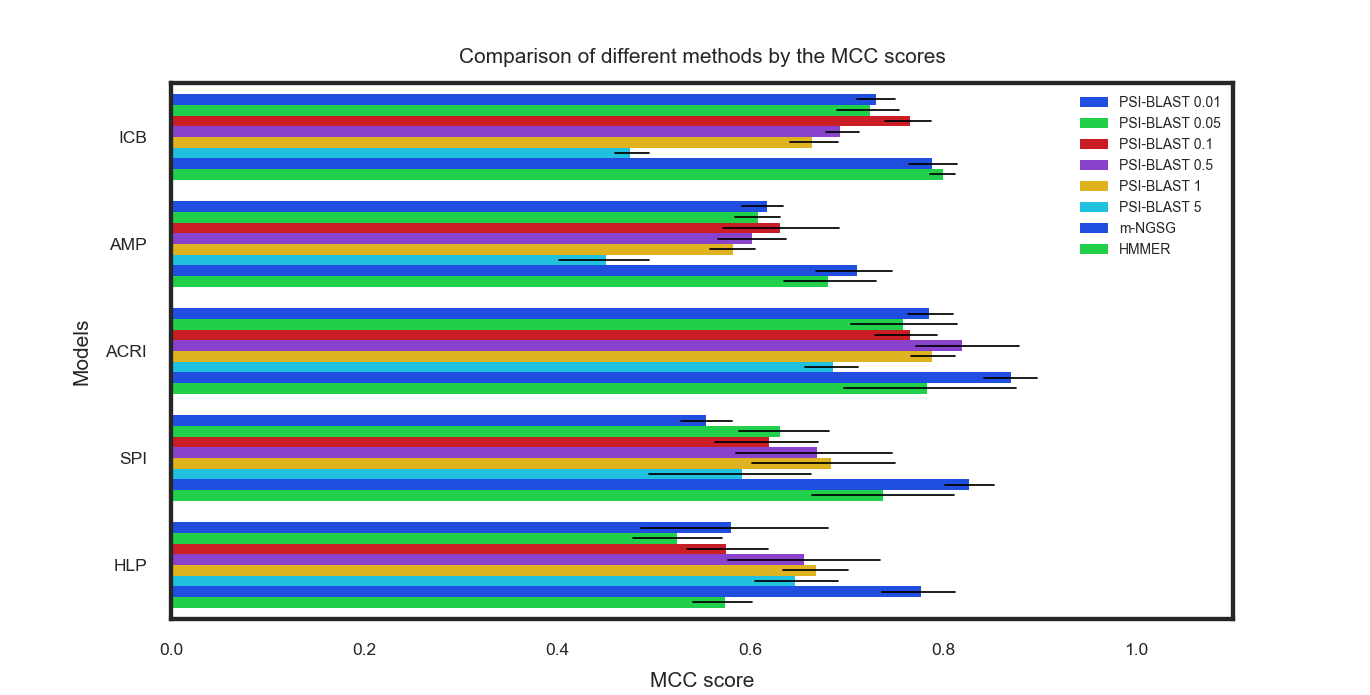


**Supplement Figure 2: Comparison of MCC.** This figure illustrates the comparison of MCC (Mathews Correlation Coefficients) among PSI-BLAST (E-value 0.01, 0.05, 0.1, 0.5, 1and 5), m-NGSG, and HMMER. The Y-axis shows different function-based models, the X-axis shows the MCC scores with their standard errors, and each bar plot shows the method used to build the models. Except for the ICB dataset, m-NGSG-based models returns better MCC scores for all other four datasets, while PSI-BLAST shows lower values for all the data sets.


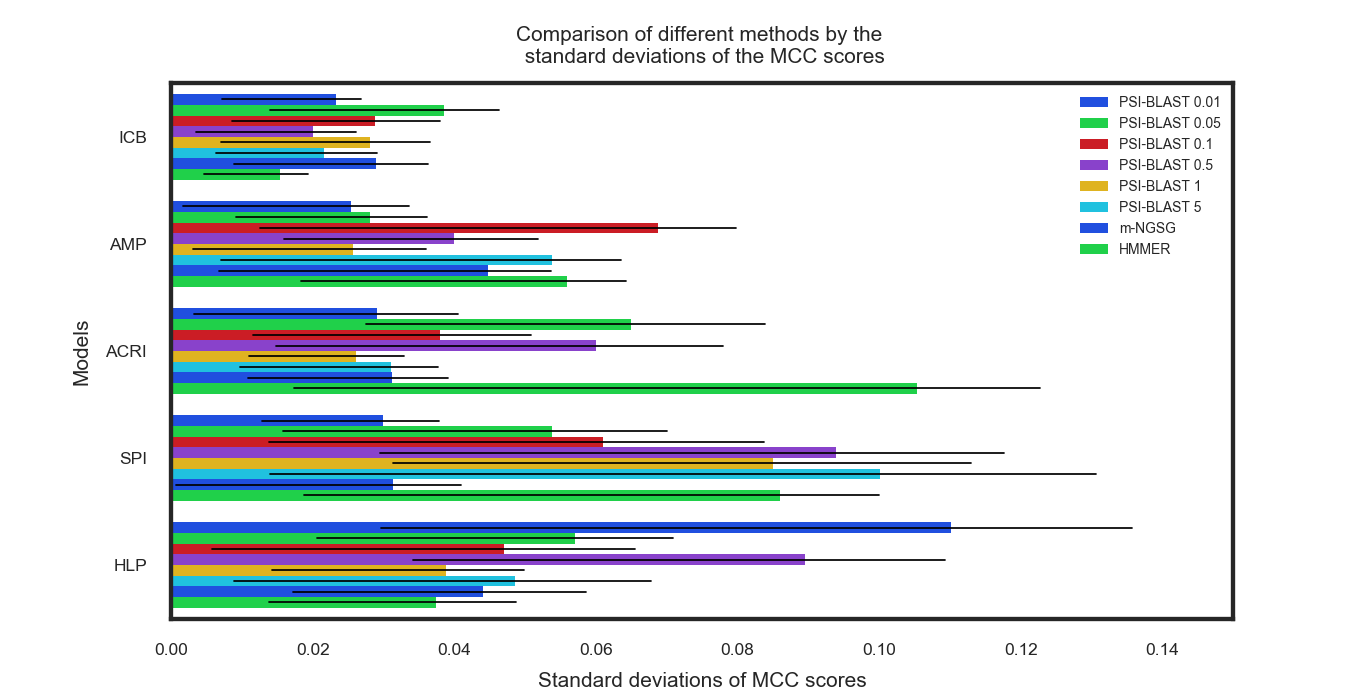


**Supplement Figure 3: Comparison of MCC Standard Deviations.** This figure illustrates the comparison of standard deviations of MCC (Mathews Correlation Coefficients) scores among PSI-BLAST (E-value 0.01, 0.05, 0.1, 0.5, 1and 5), m-NGSG, and HMMER. The Y-axis shows different function-based models, the X-axis shows the standard deviations of the MCC scores with their standard errors, and each bar plot shows the method used to build the models. This figure shows the depth of performance-consistency of each model: the higher the standard deviation, the lower the performance-consistency. The m-NGSG-based models shows standard deviations of MCC scores lower than 0.05 for each model while HMMER and PSI-BLAST shows high standard deviations for a few models.


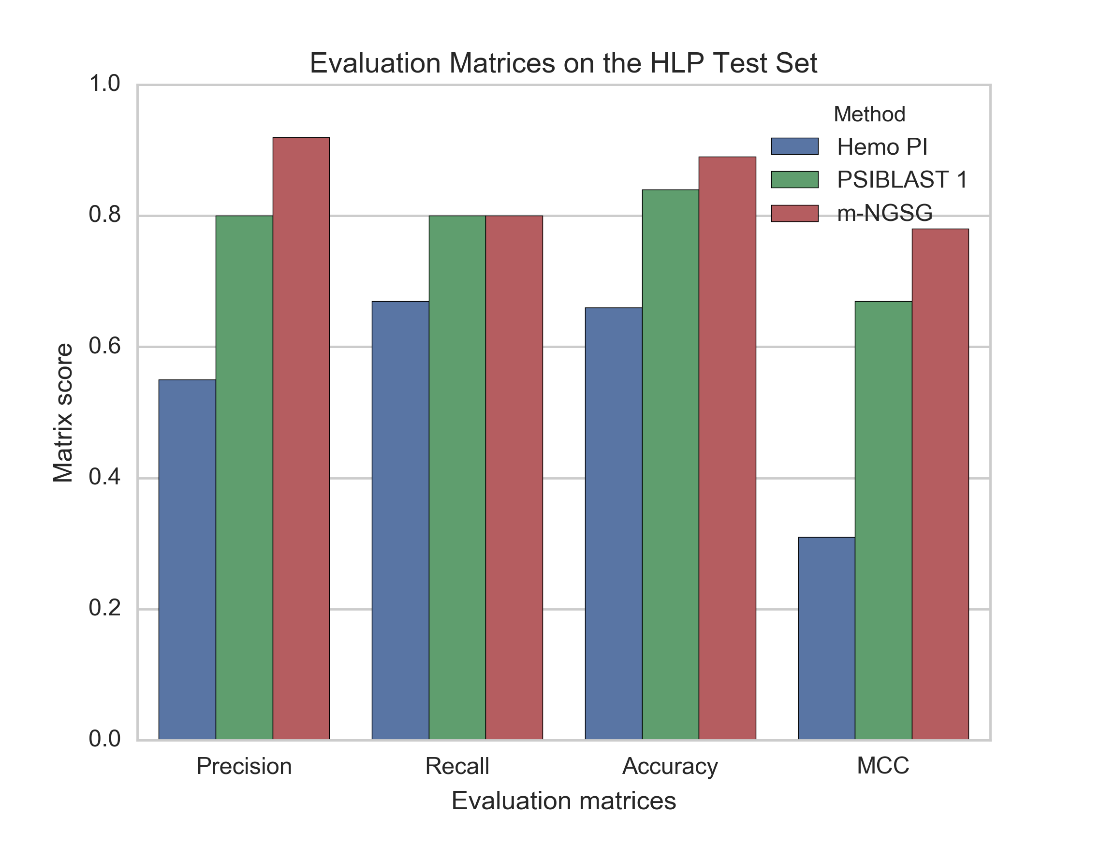


**Supplement Figure 4.** The precision, recall, accuracy and MCC values obtained applying each method on the out of sample HLP test set. The m-NGSG-based HLP model performed better than the Hemo PI in respect to each of evaluation matrices (Precision, Recall, Accuracy and MCC).


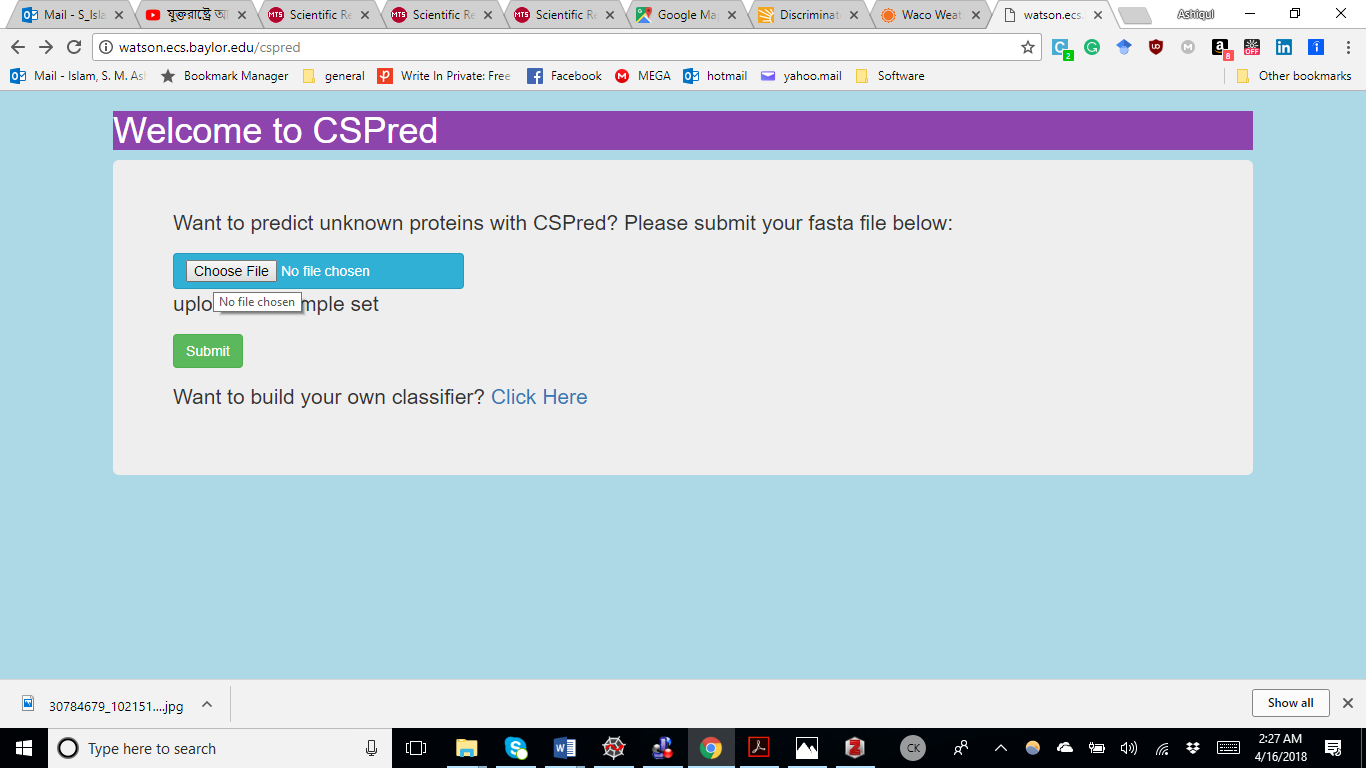


**Supplement Figure 5:** The snapshot view of the <http://watson.ecs.baylor.edu/cspred> where the CSPred web application is freely available to use. Users need to click on the grey “Choose file” button to upload the fasta file which contains the unknown protein sequences. It is recommended to input a fasta file that contains less than five-hundred sequences.


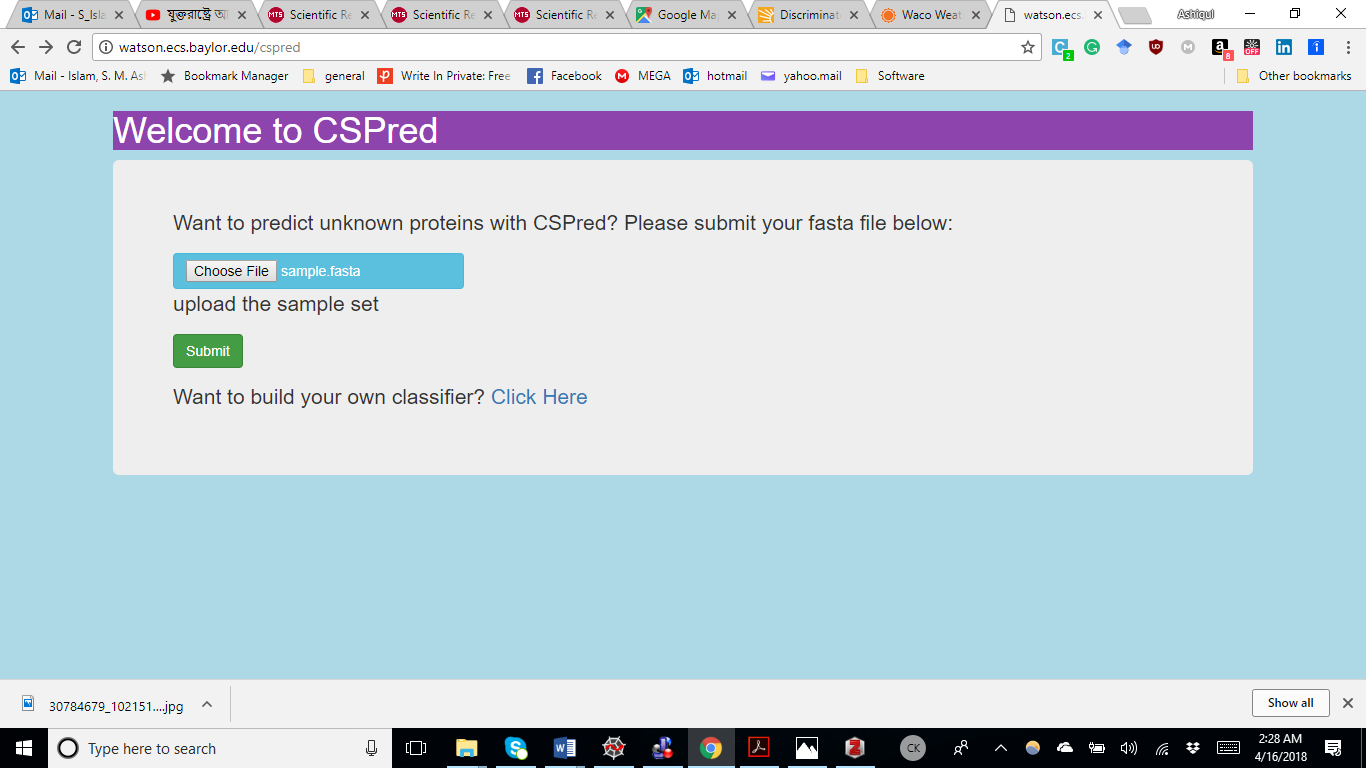


**Supplement Figure 6:** The snapshot of the CSPred page after uploading a fasta file.At this point, the file is ready to submit. The users need to click on the green “Submit” button to get the results. After clicking on the “Submit” button, the classification algorithm with start to work and it may take a while depending on the input fasta file size to display the result page.


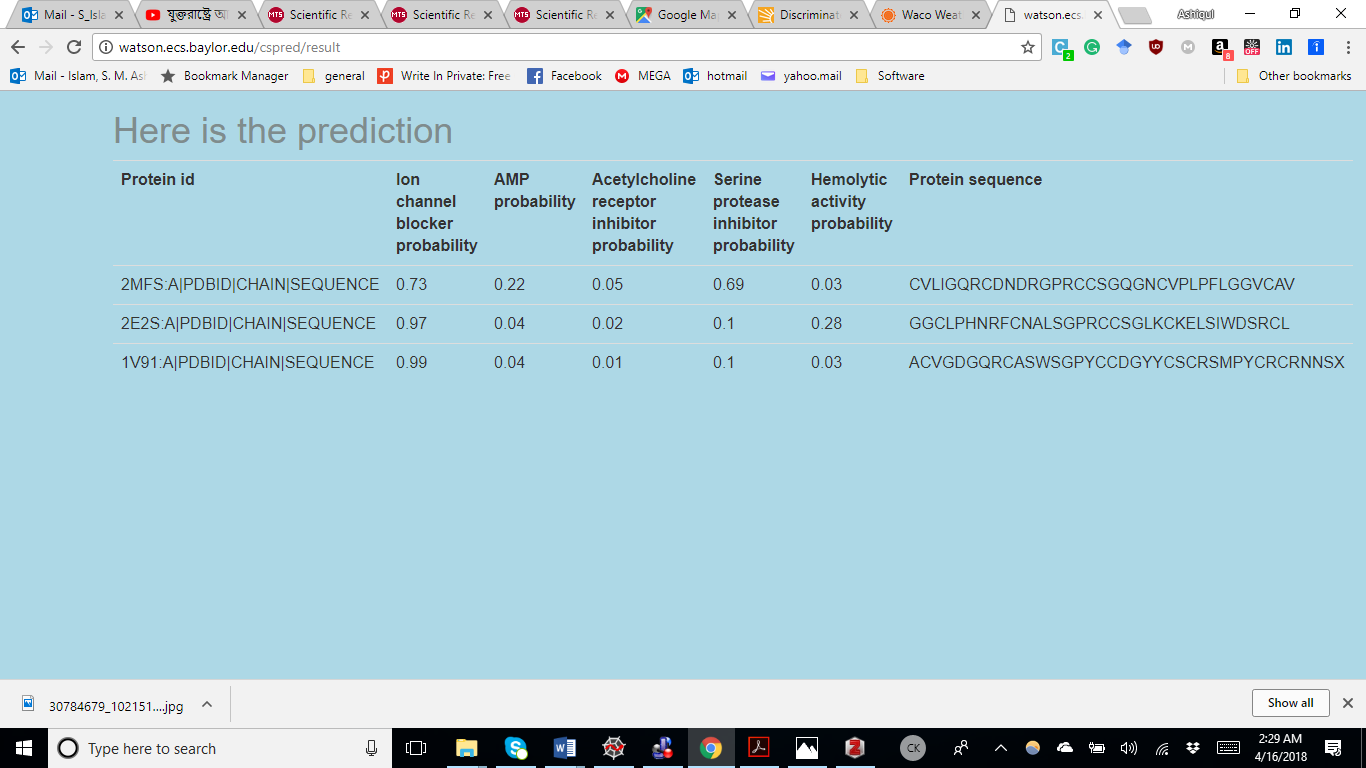


**Supplement Figure 7:** The snapshot of the result page of CSPred. The page displays seven columns. The first column shows the protein ids from the submitted fasta file, second to sixth columns show the probability value (0-1 scale) of being ion channel blocker, antimicrobial peptide (AMP), acetylcholine receptor inhibitor, serine protease inhibitor and hemolytic peptide, respectively. The seventh column form the left shows the primary sequences of each sample proteins.

1. Islam, S. M. A., Heil, B. J., Kearney, C. M. & Baker, E. J. Protein classification using modified n-grams and skip-grams. *Bioinformatics* (2017). doi:10.1093/bioinformatics/btx823
